# Heart Rate Variability in Individuals with Down Syndrome: A Scoping Review with Methodological Considerations

**DOI:** 10.3390/ijerph20020941

**Published:** 2023-01-04

**Authors:** Jakub S. Gąsior, Antonio Roberto Zamunér, Margaret Madeyska, Anna Tomik, Cezary Niszczota, Craig A. Williams, Bożena Werner

**Affiliations:** 1 Department of Pediatric Cardiology and General Pediatrics, Medical University of Warsaw, 02-091 Warsaw, Poland; 2 Departamento de Kinesiología, Universidad Católica del Maule, Talca 3480112, Chile; 3 Pediatric Cardiology and General Pediatrics Clinic, Jan Polikarp Brudziński Pediatric Hospital, 02-091 Warsaw, Poland; 4 Faculty of Medicine, Medical University of Warsaw, 02-091 Warsaw, Poland; 5 Children’s Health & Exercise Research Centre, Sport and Health Sciences, College of Life and Environmental Sciences, University of Exeter, Exeter EX1 2LU, UK

**Keywords:** Down syndrome, heart rate variability, cardiac autonomic dysfunction

## Abstract

Individuals with Down syndrome (DS) present similar heart rate variability (HRV) parameters at rest but different responses to selected movement maneuvers in comparison to individuals without DS, which indicates reduced vagal regulation. The present study undertakes a scoping review of research on HRV in individuals with DS, with special attention paid to the compliance of the studies with standards and methodological paper guidelines for HRV assessment and interpretation. A review was performed using PubMed, Web of Science and CINAHL databases to search for English language publications from 1996 to 2020 with the MESH terms “heart rate variability” and “down syndrome”, with the additional inclusion criteria of including only human participants and empirical investigations. From 74 studies, 15 were included in the review. None of the reviewed studies met the recommendations laid out by the standards and guidelines for providing the acquisition of RR intervals and necessary details on HRV analysis. Since authors publishing papers on this research topic do not adhere to the prescribed standards and guidelines when constructing the methodology, results of the research papers on the topic are not directly comparable. Authors need to design the study methodology more robustly by following the aforementioned standards, guidelines and recommendations.

## 1. Introduction

Down syndrome is a well-known complex of anomalies accompanied by the most frequent autosomal aneuploidy. It is caused by the presence of a supernumerary chromosome 21 (trisomy 21) [1]. Individuals with Down syndrome often present with various forms of congenital cardiac malformations, occurring in 44–56% of fetuses with trisomy 21 and in 40% of livebirths, most commonly in the form of an atrioventricular septal defect (AVSD) [2]. Even if there is no structural heart disease or it is not a spectrum of AVSDs (such as tetralogy of Fallot, atrial or ventricular septal defect), abnormal development of atrioventricular separating structures is connected to the embryogenesis of the cardiac conduction system and may cause spontaneous atrioventricular blocks in children and adults with this genetic syndrome [3,4,5,6]. Furthermore, different types of conduction disturbances may be observed as a natural complication of surgical procedures performed in individuals, even as a late result [7].

Several papers showed that, in comparison to peers with normal development, individuals with DS present alterations in cardiac autonomic nervous system (ANS) activity, i.e., the inability of heart rate (HR) and blood pressure (BP) to adjust accordingly in response to an activity, exercise or selected movement maneuvers [8,9,10,11,12]. Cardiac ANS abnormalities in individuals with DS could be explained by at least three possible causes. Firstly, impairment of cardiovascular functioning could be due to disturbances in brainstem areas [13] that possibly affect ANS areas [14]. Secondly, sinus node dysfunction associated with AVSDs impairing the functioning of structures of the electrical conduction system of the heart. Finally, individuals with DS are at a greater risk for the development of cardiometabolic diseases due to the overweight and/or obesity [15,16], low levels of physical activity [17], and sedentary behavior [18] apparent in this population that weakens the ANS [19,20]. Thus, cardiac ANS abnormalities in individuals with DS may be a consequence of variations within the aforementioned areas of the central and autonomic nervous system or heart, overweight and/or obesity, prolonged sedentary lifestyle or some combination of these factors.

Heart rate variability (HRV) components provide a measurement of the degree of modulation of the cardiac ANS by the use of noninvasive methods [21,22]. Autonomic imbalance, reflected by a diminished HRV [23,24,25,26], has been observed in pediatric [27] and adult patients with a variety of clinical conditions [26,28]. In 1996, standards for the measurements of HRV, as well as their physiological interpretation and clinical use were published by the Task Force of the European Society of Cardiology and the North American Society of Pacing and Electrophysiology [21]. Since that time, numerous methodological papers underlying the importance of accounting for additional crucial issues that were not a part of the Task Force Guidelines (i.e., measurement environmental conditions and respiratory rate monitoring during the short-term examination) have been published [29,30,31,32,33,34,35,36,37,38,39,40,41,42,43,44]. Therefore, researchers should follow the Task Force recommendations but also account for up-to-date important methodological advancements in measuring HRV. Recently, a systematic review showed that none of the included studies on HRV in pediatric patients with cerebral palsy (CP) met the requirements set out by guidelines related to the inclusion of details about RR interval acquisition and HRV measurements [45]. In 2018, systematic review on HRV in individuals with DS showed that this group presented autonomic dysfunction, which may or may not be expressed at rest, but it is usually demonstrated in autonomic tasks [46]. Discrepancies in HRV parameters obtained in resting conditions among DS individuals may result from implementation of methodologies that were not compliant with the standards, recommendations and methodological papers [21,29,30,31,32,33,34,35,36,37,38,39,40,41,42,43,44]. Such heterogeneity in the results of HRV studies may be caused by the limitations within the assessment and analysis of HRV [33]. 

Characterization of cardiac ANS activity in individuals with DS is important for a better understanding of the disturbances and for establishing effective therapeutic goals. The assessment of HRV studies in individuals with DS for their adherence to the published standards and methodological papers is crucial to be able to accurately summarize the current knowledge and to plan future studies. Therefore, the aim of this study was to review the existing literature on HRV in individuals with DS and to analyze the compliance of these methodology studies with the standards from 1996 and subsequent methodological articles for HRV analysis.

## 2. Materials and Methods

This study is part of a research program focused on analyzing the existing literature on HRV in patients with various diseases, intending to verify the adherence of the selected publications to the published guidelines and methodological recommendations for HRV analysis and its interpretation. The procedures concerning literature search and identification, screening, eligibility and inclusion are presented in a previously published review on HRV in pediatric patients with CP [45].

In this study, the scientific literature was screened for articles presenting HRV parameters in individuals with DS according to the updated guideline for the systematic reviews—Preferred Reporting Items for Systematic Reviews and Meta-Analyses (PRISMA) 2020 statement [47,48], with an extension designed for scoping review [49] using the PubMed, Web of Science and Cumulative Index to Nursing and Allied Health Literature (CINAHL) databases. The databases were explored using the mesh terms “heart rate variability” and “down syndrome” or “trisomy 21” and included the following limitations: (i) studies limited to humans; (ii) publications in English; (iii) publications from 1996.03.01 (publication of the standards for HRV analyses [21]) to 2020.12.31; and (iv) empirical investigations, i.e., studies involving active data collection. 

The following types of articles were excluded: meta-analyses, expert opinions, reviews and single case reports. Two researchers performed identification and screening procedures independently (November 2020). Additionally, the software StArt (State of the Art through Systematic Review) was also used to perform the identification and screening procedures (developed by the Federal University of São Carlos). An initial identification of the results of the search was performed on the titles and abstracts, with an analysis of the full texts being performed in cases that were uncertain. In an effort to expand the search, references of the selected articles were examined for studies not found in the database. Four researchers independently assessed the selected studies for eligibility. In the case of differences of opinions, the matters were discussed with the other co-authors and thusly resolved. 

By the use of the PICOS approach (patient population or disease being addressed (P), interventions or exposure (I), comparator group (C), outcome or endpoint (O), and the study design chosen (S)) [47], the following information was extracted from each study:

(1) First author and year of publication;
(2) Participant characteristics (experimental group—individuals with DS; control group);
(3) Conditions for RR interval acquisition;
(4) HRV analysis methods;
(5) Results of HRV analysis.

With the guidance of the HRV analysis guidelines, the following qualitative features of HRV studies in individuals with DS were analyzed and reviewed:

(i) Study sample (experimental and control group sizes);
(ii) The acquisition and processing of data, in which we analyzed the following:
  - Device, software, sampling frequency and duration of recordings;
  - Environmental conditions during recordings: time of day, room conditions (lighting/sounds/temperature), behaviors prior to recordings (sleep, physical activity, meals, beverages and toilet before) and heart rate stabilization;
  - Respiratory rate during recordings and breathing control;
  - Position during recordings.
(iii) HRV analysis, focusing on the following points:
  - Software, artifact/ectopic beats correction, time series length (time/beats) and data normality;
  - Parameters, bands for frequency-domain analysis and analysis method.
(iv) HRV correction for HR.

Detailed analysis and explanation of the relevance of each issue is presented in Section 4.

## 3. Results

**Figure 1:**
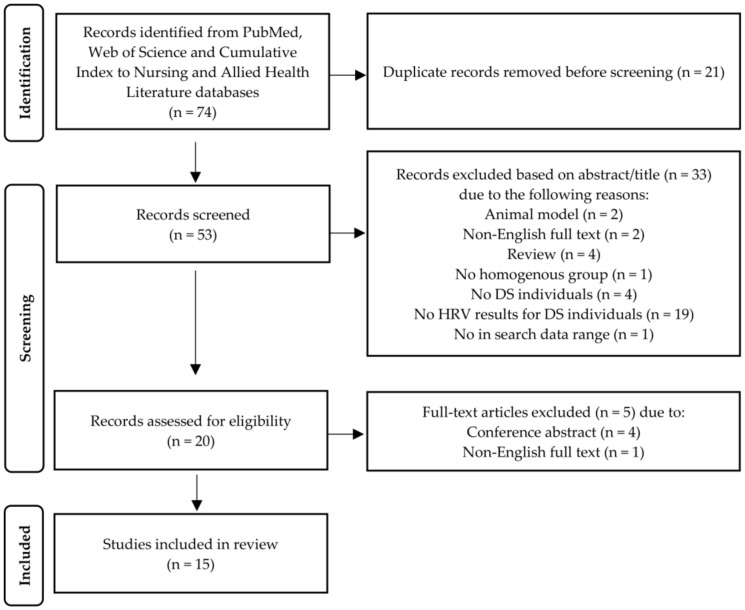
Flow diagram for the search process (PRISMA flow chart).

The data retrieved from the selected articles pertaining to points (1), (2), (3) and (4) are presented in Table 1 and Table 2, respectively (please see last pages of the manuscript). Data for point (5) are presented in Table S1 (time-domain parameters; Supplementary Materials) and Table S2 (frequency-domain parameters; Supplementary Materials). Figure 1 illustrates the search procedure.

### 3.1. Selection of the Studies

The search result generated a total of 74 articles within the database (Figure 1), from which 21 were removed due to duplication. Of the remaining 53 articles, 33 did not meet the inclusion criteria, leaving 20 to undergo a full-text analysis. Finally, 5 more articles were discarded for specified reasons (see Figure 1), leaving a total of 15 studies to be included in the systemic review [50,51,52,53,54,55,56,57,58,59,60,61,62,63,64]. One additional study was identified by checking the references within these 15 studies. However, access to the full text was restricted, despite an e-mail with the request for the article sent to the corresponding author.

### 3.2. Information Provided by the Selected Studies

#### 3.2.1. Participants/Demographic Data

Details concerning participant characteristics from the experimental (individuals with DS) and control groups are presented in Table 1. A total of 286 participants with DS aged 8–50 years participated in the analyzed studies. In one study, results of the parameters of individuals with DS and mental intellectual disability were compared with the results of individuals with DS without mental intellectual disability. The control groups comprised of a total of 191 participants aged 8–49 years described as: normally developed or without DS or healthy.

#### 3.2.2. RR Interval Recordings

Table 1 presents details on RR interval acquisition. Long-term 24 h ECG recordings were used in one study. Fourteen studies performed short-term RR interval recordings for a duration ranging from 2 to 20 min—one study did not report the recorded length of the RR interval recordings. Five studies used heart rate monitors. The range of the sampling frequency was 128 to 1000 Hz. A comprehensive depiction of the time of data acquisition, environmental conditions during recordings, a preceding period before recordings (i.e., for HR stabilization) and habits prior to the recordings (i.e., sleep routine, physical training, meal, drinks and toilet before) were not provided by any of the studies relying on short-term recordings provided. Six studies monitored and controlled the breathing rate. In 11 studies, the participants alternated between at least two different positions, i.e., seated and supine, during the short-term recordings.

#### 3.2.3. HRV Measurement

HRV measurement data are depicted in Table 2. Two studies described artifact correction as well as time series length of HRV analysis and reported the software used to calculate the HRV parameters. Details describing the verification of data distribution was presented in eight articles. One study included the results of the time- and frequency-domain analyses and a nonlinear dynamics analysis. Five studies solely provided data regarding frequency-domain parameters. In three studies, the authors presented adequate information about frequency analysis methods.

#### 3.2.4. HRV Results

Tables S1 and S2 provide the detailed raw results obtained from the reviewed studies regarding HR, time-domain, frequency-domain and nonlinear HRV parameters, respectively.

## 4. Discussion

Evaluating HRV is useful in analyzing autonomic responsiveness, as it is a representation of the autonomic influence on cardiac periodicity [43]. Even though the study of HRV was first introduced over 30 years ago, there is still hesitation towards using it as a decision-making tool in clinical practice. The limitations of HRV analysis causing misinterpretation of the data by nonexperts may be a reason for this reluctance. Thus, to overcome these issues in future studies, the limitations of HRV must be defined, and its limitations more easily disseminated and understood.

In 2018, Carvalho et al. presented the results of a systematic review and a meta-analysis on HRV in individuals with Down syndrome [46]. No significant changes were noted in most time- and frequency-domain HRV parameters obtained during rest conditions between individuals with DS and controls. Individuals with DS did present different responses to selected movement maneuvers compared to controls, which indicates a reduced vagal regulation and increased sympathetic modulation. Similar screening and inclusion criteria to review by Carvalho and colleagues [46] were implemented in the current study. Therefore, we analyzed most of the articles assessed by Carvalho et al. but with a focus on methodological aspects of HRV analysis, including details concerning data acquisition and HRV measurements. However, it was found that the majority of the studies either did not adhere to the standards and guidelines regarding RR recording and HRV analysis or the information about the applied methodology was limited. Both of these reduced the ability to reproduce the studies consistently. However, over the last years, more information has been provided about data acquisition and HRV analysis and, thus, some improvements in reporting have been demonstrated. As shown in Table 2, the included studies published from 2013 onwards presented detailed information for most of the outcomes considered in this systematic review, with only one study that did not report information on “artifact correction” and another one that did not report information on “time series length”. Indeed, details regarding “time series length” was the most missing information in the included studies (33%). 

HRV results can be significantly influenced by uncontrolled variables within the environment [33]. To ensure that researchers are able to produce accurate and reproducible results, precise protocols must be built by carefully evaluating physiological, environmental and contextual factors, such as: the time of day in which RR intervals are recorded; participant variables, such as age, sex, HR, breathing rate, health and physical activity status, medications, food and water consumption or voiding prior to recordings; body position during short-time recordings; signal quality (length of recording period, method of recording or detection, sampling frequency and breathing—paced or free breathing); laboratory room settings (i.e., temperature, lights and noise control). Details on HRV analysis tools should also be precisely provided (i.e., methods of HRV metrics calculation—software, artifacts removal, frequency-band cutoffs and power spectral analysis method) [32,33,36,38,65,66].

Compliance of the studies to standards, recommendations and guidelines are presented below in appropriate paragraphs.

### 4.1. Study Sample

There were a total of 286 participants with DS across the studies that met the inclusion criteria—the lowest number of participants in the experimental group was 7, the highest—50 individuals. Control groups were comprised of 191 participants. The number of individuals in the control groups ranged from 6 to 25. 

Quintana [67] stated that HRV studies are generally underpowered, a phenomenon not uncommon in physiological studies. In case–control studies focusing on vagally mediated HRV measures, detection of small (effect size of 0.25), medium (effect size of 0.5) and large (effect size of 0.9) effect sizes requires samples of 233, 61 and 21 participants, respectively (significance criterion of alpha = 0.05), in order to obtain 80% statistical power [38,67]. Thus, in order to confirm results from previous studies with a smaller sample size, larger populations of DS participants need to be included in future investigations. 

### 4.2. Data Acquisition and Processing (Device, Duration of Recordings and Sampling Frequency)

Details regarding the software used to record the raw data and generate the RR intervals were included by all of the authors of the studies included in this review: an ECG device was used in nine articles; five articles used a heart rate monitor; and, in one study, authors used a finger photo-plethysmography. 

HRV studies should include information on the signal acquisition devices used and the software applied for the generation of the RR intervals and statistical analysis. If this information is unavailable, reproducibility can be maintained if the authors provide precise methodological specifications [34]. Traditionally, the electrocardiogram (ECG) is used to record the RR interval data but it can also be performed using more recent methods, such as heart rate monitors [68], and photo-plethysmography (PPG) [34,37] or mobile applications with a chest strap [69,70]. Even though there is about a 6% discrepancy between ECG and PPG for most HRV measurements [71], certain PPG devices in some populations have had satisfactory agreement with ECG [72,73,74]. When comparing ECG to PPG or mobile applications, ECG is more accurate in identifying and correcting artifacts and ectopic beats, as well as in correctly identifying cardiac events, such as atrial fibrillation or arrhythmias [34,38,75,76].

Obtaining RR intervals in individuals with DS in certain situations, such as during stable conditions before or after the intervention, is achievable with a traditional ECG. However, recording a viable ECG during exercise may be problematic in this population and the use of heart rate monitors could be a feasible solution. Heart rate monitors have been confirmed as an acceptable method for recording HRV [77] and detecting RR intervals [68] in the healthy population. However, data quality of RR intervals recorded using heart rate monitors or mobile applications with a chest strap would likely be more problematic in individuals with DS because ECG abnormalities are more frequent [78]. However, in such circumstances, autonomic assessment during provocations might be of clinical value. One possible solution could be to choose heart rate monitors that provide access to raw data; however, it is not the same as monitoring the ECG in real time and one cannot recreate an ECG from R–R interval data. Consequently, the quality of the heart rate monitor recording must be questionable. In the future: i) the first recording should be performed using ECG to detect potential abnormalities, and ii) studies should be conducted to verify whether heart rate monitors or mobile applications with chest straps are able to reliably detect RR intervals and, thus, properly record HRV (for details, see [79]) in the DS population under varying conditions.

Signal recording duration and RR interval length used to calculate HRV parameters represent different information and should be stated clearly and separately. Only one study included in this review recorded 24-h ECGs. The authors of this study did not state what period of time was used to analyze NN intervals. In other studies, the recordings ranged from 2 to 20 min in duration. Ten studies provided both the duration of the recording (or number of points of RR series) and the length of the RR interval series used to calculate the HRV parameters. It is important to include the number of points of the RR series, as this parameter is equally a function of time and HR, and certain methods of HRV analysis (e.g., nonlinear approaches) depend upon the length of the RR series. 

Sampling frequency was provided in 11 studies (from 128 to 1000 Hz), which is an especially important parameter due to its direct effect on the resolution of the RR intervals, and limits the evaluation of HRV and should, therefore, be provided. The validity of the HRV parameters is decreased when using lower signal sampling rates (i.e., <250 Hz), mostly for nonlinear indices and frequency-domain parameters [21,36,37,38,80]. Applying appropriate interpolation algorithms may enable the use of sampling rates between 100 and 250 Hz [37]. Task Force experts recommend using sampling rates below 500 Hz but above 250 Hz, while other researchers advise using sample rates from 500 Hz to 1000 Hz [38]. 

### 4.3. Environmental Conditions during Recordings: Time of Day, Room Conditions (Lightings/Sounds/Temperature), Behaviors Prior to Recordings (Sleep, Physical Activity, Meals, Beverages and Toilet before) and Heart Rate Stabilization

When performing experiments, especially those focused on short-term (5 min, 1 min, 10 s) HRV, it is important to control environmental and external factors [32,33,38,65]. A full account of the environment during the recordings—the time of day, room conditions and behaviors of the participants before the recording—was not provided in any of the reviewed studies regarding short-term recordings.

Only Mendonca et al. provided almost all details of the experimental conditions in their studies [58,59,60]. Wherever possible, environmental conditions should be regularized for all participants of the study, and a detailed description always provided. A useful demographic questionnaire was provided by Laborde et al. (2017) [38] that was helpful in collecting and controlling possible HRV confounding variables [38]. 

Eight studies stated the exact parameters for planned HR stabilization before the start of data recording, lasting from 5 to 15 min. HR is typically uneven, fluctuating throughout the day and with changes in position [33,81]. Therefore, time must be allocated for the volunteers to adjust to the recording process to allow for stabilization of the HR, thus making the analysis of short-term HRV possible. This acclimatization process usually comprises a 5- to 10-min rest period before the commencement of the experiment [34,37,38]. When baseline HRV is being recorded, it is important for the participants to remain still and silent [38]. Speaking causes a respiratory sinus arrhythmia and asking the participants to remain silent ensures the elimination of this respiratory component of HRV. None of the studies reported to have asked participants to not talk or remain still during data registration. In four studies, spontaneous breathing was reported. In two studies, authors monitored breathing rate.

### 4.4. Respiratory Rate during Recordings and Breathing Control

Respiratory diseases (e.g., obstructive sleep apnea [82], pneumonia [83], pulmonary hemosiderosis [84] and cystic lung disease [85]) and/or problems (e.g., abnormalities of the airways and lungs, glue ears and recurrent lower respiratory tract infection [86]) are a common cause of morbidity and mortality in individuals with DS [87]. Case studies have shown that children with DS may present with a respiratory rate of 43 [88], 51 [89] or even 60 breaths per minute [85]. Breathing frequency and depth affects HRV (mostly frequency-domain parameters) [33,90,91]. A breathing rate of ~3 to 9 and 9 to 24 breaths per minute affects the low- and high-frequency bands (LF and HF, respectively) of HRV, respectively [36,38,40]. The investigator should be aware that the spectral powers at LF and HF bands are associated with the sympathetic and parasympathetic and vagal influences on HR, respectively, and that changes in respiratory rate may, therefore, nullify the spectral indices.

Młyńczak and Krysztofiak stated that breathing depth may be more important than rate [92]. The authors have also proposed cardiorespiratory temporal causal links with the path for lying supine from tidal volume, through heart activity variation and average heart activity, to respiratory timing [92]. The method used to measure tidal volumes (e.g., by using a mouth piece) could affect HRV data [93]. This issue could possibly be resolved in future studies by using the Pneumonitor 2 or 3 (a portable device, which records breathing patterns) along with a single-lead ECG, motion and/or pulse oximetry (saturation, pulse wave) [94,95].

Four HRV studies in individuals with DS reported that breathing rate was spontaneous. A very important question is whether changes in a given parameter (e.g., respiratory rate) have a greater effect on changes in HRV parameters versus others, such as HR [96]. Changes in HRV in response to changes in breathing pattern are to be expected, which is why knowing the respiratory rate is required to be able to validate whether the primary cause of the changes in HRV are due to respiratory rate changes [38]. This is especially pertinent when an experimental task changes the normal respiratory pattern, as well as in populations that naturally have an altered breathing pattern [33,34].

In HRV studies, an optimal solution has yet to be found for how to control and record the respiratory rate [33]. Quintana and Heathers proposed to measure the participants’ natural respiratory rate and using that data from the resting state registration as a form of rate pacing [33]. Another solution is to stabilize the respiratory rate and HR by allowing the participant to adjust to the recording environment. This is accomplished by asking the participant to take up the position in which the recording will take place and remaining in that position for an allotted amount of time before the recording is started. The respiratory rate can then be derived from the ECG with the application of the proper algorithms [97] or by the simultaneous use of a respiratory belt with the ECG. There have also been cases when a camera has been used to monitor and record respiratory rate in individuals where the RR interval is being recorded by an HR monitor [98]. It should also be noted that changing the HRV frequency bands or excluding participants who have a respiratory rate that is outside of the established ranged would be justified. Finally, the previously mentioned Pneumonitor, a device that provides cardio-respiratory measures, could be used. It is also important for researchers to take into account that the choice between a participant’s breathing being spontaneous or paced will impact the HRV analysis in different ways [99,100,101,102,103].

### 4.5. HRV Analysis

#### 4.5.1. Software, Artifact Correction, Time Series Length (Time/Beats), Information about Data Normality

A complete depiction of the information regarding the software employed in the calculation of the HRV parameters, time series length used in HRV indices calculations, and methods of artifact correction were only presented in two out of the fifteen reviewed articles. 

Artifacts recorded during data acquisition may have a significant impact on the HRV parameters [29,36,104,105,106,107,108,109,110]. Prior to calculating HRV parameters, the raw data should always be preprocessed and the information for this step described. The recorded data should be analyzed to identify and process artifacts (e.g., false or missed beats) as well as all beats that are not generated by the depolarization of the sinus node (i.e., abnormal beats). This process of reducing/replicating and cleaning the data should be described fully and validated [29,32,34,37,42,111,112,113].

Kubios HRV software, mostly used to process HRV recordings, provides tools for handling artifacts, i.e., correction method—threshold-based artifact correction [114]. Recently, Alcantara et al., showed that the application of different Kubios filters has a significant impact on HRV-derived parameters obtained from short-term recordings in both time and frequency domains [107]. Therefore, the authors stated that it should be mandatory to report the Kubios filter used [115].

In studies that utilize protocols reliant on sample numbers, methodological relevance is placed on RR interval length and HR series [37]. Various durations have been proposed, by various groups of authors, required for the collection of reliable time- and frequency-domain parameters [36,37]. They have also proposed different nonlinear approaches [116,117,118]. Concurrently, in accordance with the Task Force paper [21], a common recommendation for the assurance of comparability between papers is for linear HRV short-term parameters to be calculated from 5-min intervals [38]. The choice for series length for nonlinear calculations depends on the method employed. It is reasonable to follow the recommendations of the original paper to allow for a comparison between the studies.

Statistical analysis must also be considered. Of the articles reviewed in the present paper, eight described the process of checking the data distribution. In another two studies, a logarithmic transformation was performed. The majority of parameters in many HRV studies presented with a non-normal distribution; hence, to meet statistical requirements, it is necessary to apply nonparameter tests or a log transformation, then present and evaluate the resulting data [38,119].

#### 4.5.2. Frequency-Domain and Nonlinear Parameters

The most commonly used frequency-domain parameters in HRV studies are LF and HF powers, used with 0.04–0.15 Hz and 0.15–0.40 Hz bands, respectively, and, subsequently, the LF/HF ratio [21,37,42]. However, for a breathing rate of over 24 breathes per minute (common for children), the upper limit of the HF band should be increased to adequately set the HF range [34,36,38]. The recommended HF band width for this population is 0.24–1.04 Hz at rest, as proposed by Quintana et al. [34].

The most commonly used methods for the analysis of frequency-domain HRV are the autoregressive (AR) spectral and fast Fourier transform (FFT) analyses [42]. An explanation of the chosen frequency-domain method and any methodological assumptions should be provided, such as windowing method, window length and overlap. This is due to the fact that, in healthy subjects at rest, the results from these analysis methods are not interchangeable [120]. In the present review, 13 of the studies reported the chosen analysis method for obtaining the frequency domain parameters (FFT: 4, AR: 9), with no study describing the sampling and windowing.

Frequency-domain HRV parameters can be expressed in absolute (ms2), relative or normalized (nu) power [21,36]. Values should be presented in both units [38] to avoid the limitations encountered when only using one [32]. Whereas the absolute power of the HF band can be considered a robust index of cardiac vagal regulation, LF band absolute power cannot be considered a clean index of cardiac sympathetic regulation. The representation of a robust index of cardiac sympathetic modulation by the normalized power of the LF band has been shown by some authors with the use of autonomic maneuvers [120]. Powers were presented in both units in three of the studies and only in absolute units in six of the studies. 

Only three studies presented results from methods of nonlinear dynamics—approximate entropy, correlation dimension, detrended fluctuation analysis (short-term) and symbolic dynamics. In 2015, on behalf of the European Heart Rhythm Association, a review was performed by Sassi et al., examining the most commonly used nonlinear approaches and their impact on the understanding of HRV [121]. Recently, in 2020, a description of the nonlinear methods along with their most notable applications was published [122]. For the characterization of physiological and pathophysiological conditions, it is important to use a combination of multiple HRV parameters, as the extraction of the complete complexity of the physiological systems is not possible with the use of just one method (linear or nonlinear) [123].

#### 4.5.3. Correction for HR

The significant correlation between mean HR and HRV parameters is not accounted for in the majority of HRV studies. HRV values may be determined, to some extent, by differing HR values, which have differing impacts on HRV [31,35,124,125]. Some authors have suggested to remove the HRV dependence on HR before deductions are made regarding HRV changes [126]. It is of note that HRV indices are not all dependent on the mean HR [127]. In the recent discussion of such an approach, it was recommended to always examine and describe any relationship between the HRV parameters and the predominant heart period [41]. HR values were provided in 8 of 15 analyzed studies.

### 4.6. Summary—Implications and Applications for Future Studies

For the proper interpretation of results, their reproducibility and their application into clinical practice, all possible confounding factors must be controlled and all methodological aspects must be reported in the HRV study. Insights and comparisons between the reviewed studies and different research are likely limited due to the lack of important information and the methodological heterogeneity, which could also account for some of the controversial results. These findings then produce uncertainty on how these results translate into clinical settings, thus impacting the health care professionals working with this population. This, in turn, causes HRV to be underutilized, despite the important clinical markers it provides, and questions its clinical applicability [128]. Therefore, RR recording and HRV analysis should follow the standards and methodological guidelines and be correctly reported, thereby increasing the confidence of clinicians and their healthcare teams. 

Authors of future studies on HRV in individuals with DS should:

(1) Before the RR intervals acquisition (planning): a) indicate one clinician/researcher to acquire and, more importantly, analyze data [129,130], (b) if using heart rate monitors, perform primarily ECG recordings to determine the presence of sinus rhythm and potential cardiac abnormalities in the study population, (c) if not validated, assess the validity of the device to acquire RR intervals or use a reliable device offering adequate sampling frequency (≥500 Hz), (d) include a larger study population, (e) inform participants and/or their parents/legal guardians in details about adequate behaviors prior to measurement day(s) (sleep, physical activity, meals, beverages and toilet before), (f) in case of baseline recordings, ensure adequate time for HR and respiratory rate stabilization, and (g) in case of short-term recordings, determine the position during the measurements and recording time needed to obtain adequate time series, assess and control environmental conditions (time of day, room conditions: lighting/sounds/temperature/humidity);

(2) During the RR intervals acquisition (performing): (a) in case of short-term recordings, monitor and, if possible, record respiratory rate (breathing pattern) during recordings;

(3) After the RR intervals acquisition (analyzing): (a) in case of ECG data, consult the results with an experienced cardiologist to exclude individuals with cardiac abnormalities, (b) assess and process the raw data (artifact/ectopic beats correction) before calculating HRV parameters, (c) use HRV analysis software (e.g., Kubios HRV [131] or PyBioS [132]) that provides the user the ability to set the preferences and analysis options and inform about preferences used to calculate HRV parameters: sample length, RR time series interpolation rate, RR interval detrending method (with necessary smoothing parameter if possible) and analysis options (time-domain, frequency-domain: frequency bands and spectrum estimation options, nonlinear), and (d) assess HRV data normality, (e) examine and describe any correlation between the HRV parameters and HR.

### 4.7. Limitations

Authors of this review assessed the quality of the included papers, taking into account standards for the measurements, physiological interpretation and clinical use of HRV published by the Task Force of the European Society of Cardiology and the North American Society of Pacing and Electrophysiology in 1996 [21], as well as other numerous methodological papers published in the last decade [29,30,31,32,33,34,35,36,37,38,39,40,41,42,43,44,133]. The selection of methodological papers was subjective and lack of agreement between different organizations for a standardized approach can be interpreted as a limitation of the review.

## 5. Conclusions

To summarize, there were no significant changes noted in most time- and frequency-domain HRV parameters at rest between individuals with DS and the control group. Reduced vagal regulation and increased sympathetic regulation was proposed to be the difference in response to selected movement maneuvers between the control group and individuals with DS. However, replication of the experimental design in laboratories or a clinical setting was not possible due to missing information about data acquisition and HRV measurement. Thus, the confidence in their interpretation is limited due to the absence of important methodological information [33]. Methodological misinterpretations and limitations can not only be identified, but also eliminated by following the standard and methodological guidelines. Authors should also follow recently developed HRV metrics when creating future studies regarding HRV in the DS population and should also be aware of controversies of physiological interpretation and meaning of HRV parameters [134,135,136,137,138,139,140,141].

## Figures and Tables

**Table 1:** Baseline characteristics of study participants and details concerning RR intervals acquisition.

First Author and Year of Publication	Experimental Group	Control Group	RR Intervals Acquisition				
			Software for RR Intervals Acquisition, Sampling Frequency and Duration of Recordings	Time of the Day and Room (Lights/Voices/Temperature)	Behaviors before Data Acquisition (Sleep Routine, Physical Activities, Meal, Drinks, Toilet before) and Instructions Given. Rest or Heart Rate Stabilization before Recordings	Respiratory Rate (Breathing Control) during Recording	Position during Recordings
Ferri et al., 1998 [49]	7 DS children Age: 13.9 years (range: 8.6–16.5) Additional characteristics: BMI	6 normally developed children Age: 12.8 years (range: 8.0–17.5)	Software: ECG: Oxford MPA-II recorder Sampling: 128 Hz Duration: 10 min	Subjects slept in laboratory for two consecutive nights, the recording of data carried out during the second night.	Measurements carried out during sleeping.	NR Respiratory pauses and oxygen desaturations detected automatically by the software Oxford Medilog 9200 System.	During sleeping
Baynard et al., 2004 [50]	16 individuals with DS (10♂) with mental retardation (MR) Age: 20.8 ± 0.9 years Additional characteristics: height, weight, BMI, VO^2^ peak, RER peak, VE peak	15 patients with MR (8♂) Age: 19.7 ± 2.3 years	Software: HRM: Polar Electro Oy, Kempele, Finland Sampling: resolution 1 ms Duration: 5 min seated rest; 4 min submaximal exercise stages	NR	Participants familiarized with the laboratory setting, treadmill walking, and use of the headgear and mouthpiece. Participants: rested quietly in a seated position for 5 min; performed 4 min submaximal exercise stages on a treadmill (treadmill protocol individualized); asked not to eat or drink caffeinated beverages 4 h before testing. 5 min	NR Oxygen consumption measured during the entire exercise period.	Seated (5 min) and 4 min submaximal exercise stages on a treadmill.
Figueroa et al., 2005 [51]	13 individuals with DS (8♂) Age: 27.8 ± 8.1 years Additional characteristics: height, weight, BMI, maximal grip strength	14 without DS (6♂) Age: 26.4 ± 7.5 years	Software: one lead ECG (BIOPAC) Sampling: NR Duration: 2 min period at rest, handgrip strength test and recovery	NR	Participants underwent laboratory familiarization with testing procedures prior to data collection. Participants: - tested in a post-prandial state (~3 h) and refrained from vigorous exercise 24 h before the testing; - asked to refrain from caffeine ingestion on the testing day. 5 min	NR Participants: breathing spontaneously; instructed to refrain from holding their breath and avoid Valsalva maneuver during the handgrip strength test	Seated, sustained handgrip at 30% MVC
Iellamo et al., 2005 [52]	10 individuals with DS (4♂) Age: 26.3 ± 2.3 years Additional characteristics: BMI	10 healthy volunteers (4♂)Age: 26.1 ± 4.0 years	Software: ECG—precordial chest lead (Biopac System) Sampling: 300 Hz/channelDuration: 10 min	Experiments performed in the morning in a laboratory at ambient temperature (22–24 °C).	Participants required not to eat or drink coffee for at least 2 h. The participants lay in a room made dark and noiseless. After instrumentation, the subjects lay supine for 15 min before experiments to relax (dark room, noiseless).	Respiratory signal recorded by means of a thoracic belt (Biopac). Respiratory spectra used to assess the main respiratory frequency and to locate the respiratory component of the power spectral analysis of RR interval variability.	10 min of supine rest followed by 10 min of active orthostatism.
Goulopoulou et al., 2006 [53]	50 individuals with DS (27♂) Age: 24 ± 0.9 years Additional characteristics: height, weight, BMI, VO^2^ peak	24 healthy controls (12♂) Age: 26 ± 1.1 years	Software: Modified CM5 ECG lead (Biopac Systems, CA, USA) Sampling: 1000 Hz Duration: 5 min	NR	Participants tested 4 h after their last meal and asked to refrain from exercise 24 h prior to testing and from caffeine ingestion on testing days. Prior to data collection, participants familiarized with all testing procedures. Sessions continued until each participant could comfortably walk on a motorized treadmill. 5 min	Breathing rate was visually monitored and averaged between 14 and 18 breaths per minute.	Rest and treadmill exercise test
Agiovlasitis et al., 2010 [54]	26 DS individuals (18♂) Age: 26.5 ± 7.6 (16–40) years Additional characteristics: height, weight, BMI	11 individuals without DS (5♂) Age: 25.5 ± 7.3 (17–39) years	Software: Finger photo-plethysmography (Portapres, TNO Biomedical Instrumentation Amsterdam, The Netherlands) Sampling: 200 Hz Duration: 5 min	NR	No food for at least 4 h prior, no caffeine or exercise for 24 h prior to testing. 5 min	NR	Supine and 80° head-up tilt using a tilt table
Giagkoudaki et al., 2010 [55]	10 DS individuals (4♂) Age 24.2 ± 5.1 years Additional characteristics: height, weight, BMI	10 healthy sedentary individuals (5♂) Age 23.3 ± 4.6 years	Software: 3-channel ECG Holter recorder with WinTer Holter Analyzer software (Galix Biomedical) Sampling: NR Duration: 24 h	Assessments made at baseline and after 6 months. 6-month exercise-training program conducted 3 times per week and lasted 60 min, led by three expert exercise trainers.	Participants asked to avoid caffeine and alcoholic beverages, any activity other than their daily activities that could affect heart rhythm, during the recording, to abstain from exercise when HRV data were collected at the beginning of the study and after the 6-month exercise-training program.	NR	24 h ambulatory ECG Holter
Agiovlasitis et al., 2011 [56]	16 DS individuals (8♂) Age: 26 ± 8 years Additional characteristics: height, weight, BMI	16 individuals without DS (8♂) Age: 26 ± 7 years	Software: ECG CM5 configuration (Biopac Systems, Goleta, CA, USA) Sampling: 1000 HzDuration: 10 min rest and 10 min upright tilt	NR	Participants familiarized with the experimental procedures, refrained from food for at least 4 h and from caffeine and exercise for 24 h prior to testing. 10 min in the supine position	NR	Supine and 80° head-up tilt
Mendonca et al., 2011 [57]	13 individuals with DS (9♂) Age: 34.9 ± 1.1 years (27–48) Additional characteristics: height, weight, BMI, VO^2^ peak, RER peak, VE peak, VO^2^VT	12 individuals without DS (8♂) Age: 34.8 ± 2.0 years (27–48)	Software: HRM: Polar RS 800 G3 Heart Rate monitor (Polar Electro, Kempele, Finland) Sampling: 1000 Hz Duration: 5 min	Tests carried out in the laboratory with temperature between 21 and 24 °C and a relative humidity between 44 and 56% between the hours of 7.00 and 11.00 h at approx. the same time of day for all individuals. Visits minimum of 2 days apart and a maximum of 7 days apart.	Participants familiarized with the laboratory setting, treadmill protocols, and use of the headgear and face mask. Participants asked to abstain from caffeine and vigorous exercise for 24 h prior to testing and be at least 3 h post-prandial before testing. 5 min	NR Expired gas measurements made using a computerized on-line breath-by-breath system (Quark b2, Cosmed Srl-Italy)	Seated rest, standing, submaximal treadmill exercise, standing post-exercise recovery
Mendonca et al., 2011 [58]	14 individuals with DS (10♂) Age: 35.1 ± 7.8 years (18–50) Additional characteristics: height, weight, BMI, VO^2^ peak, RER peak, VE peak	12 individuals without DS (8♂) Age: 36.0 ± 7.7 years (20–49)	Software: HRM: Polar RS 800 G3 Heart Rate monitor (Polar Electro, Kempele, Finland) Sampling: 1000 Hz Duration: NR	As above [57]	As above [57]	NR Expired gas measurements made using a portable mixing chamber system (MetaMax® I, Cortex, Leipzig, Germany)	Standing rest, submaximal treadmill exercise, standing post-exercise recovery
Mendonca et al., 2013 [59]	13 individuals with DS (10♂) Age: 36.5 ± 1.5 years (27–50) Additional characteristics: height, weight, BMI	12 individuals without DS (9♂) Age: 38.7 ± 2.4 years (27–50)	Software: HRM: Polar RR Recorder, Polar Electro, Kempele, Finland) Sampling: 1000 Hz Duration: 10 min	Participants evaluated pre- and post-training periods: first performed a treadmill, second rested position on a bed in a quiet, semi-dark environment. Tests carried out in the laboratory with a controlled temperature (21–24 °C) and humidity (44–56%).	Participants tested in the postprandial state (12 h) and asked to refrain from caffeine and exercise for 24 h before testing. After the 12 weeks of training, all participants repeated the testing procedures under the same conditions and at the same time of day. Participants asked to remain quietly without speaking or making any movements for 15 min.	Spontaneous breathing conditions. To control for the stability of breathing rate and tidal volume, participants monitored during the 10 min of supine rest using a portable mixing chamber system (Metamax1 I, Cortex, Leipzig, Germany).	First visit—treadmill graded exercise test. Second visit (48 h after the first visit) rested while lying down in the supine position on a bed.
Bunsawat et al., 2015 [60]	26 persons with DS: not matched for HR change (*n* = 11, 6♂)—age: 28 ± 3 years; matched for HR change (*n* = 15, 8♂)—age: 25 ± 2 years; Additional characteristics: height, weight, BMI, VO^2^ peak, RER peak, VE peak	15 persons without DS (6♂) Age: 27 ± 2 years	Software: single-lead ECG (BIOPAC, Santa Barbara, CA) Sampling: 1000 HzDuration: 5 min in supine (the last 5 min of the 10 min period) and 5 during an 80° head-up tilt	The passive upright tilt performed on the second day in the morning at ambient temperature (22–24 °C)	Participants in the postprandial state for 4 h and refrained from caffeine and exercise for 24 h before data collection on each testing day. The first day of testing consisted of a maximal exercise test on a motorized treadmill.	NR	Supine position for 10 min and 5 min 80° head-up tilt
de Carvalho et al., 2015 [61]	25 individuals with DS (16♂) Age: 8.6 ± 1.4 years Additional characteristics: height, weight, BMI	25 individuals without DS (16♂) Age 9.1 ± 1.2 years	Software: HRM: Polar RS800 CX monitor, Polar Electro OY, Kempele, Finland Sampling: NRDuration: 20 min	Data collected under controlled temperature (21–23 °C) and humidity (40–60%). Evaluations between 8:00 am and 11:00 am. Parents/guardian of the children stayed in the room, during all protocol.	Participants instructed to avoid consuming caffeine for 24 h before evaluation.	Spontaneous breathing	Supine
Bunsawat et al., 2016 [62]	HGS test study: 10 subjects with DS (6♂) Age: 26 ± 3 years Submaximal cycling exercise (SCE test): 9 subjects with DS (9♂) Age: 30 ± 2 Subjects with and without DS were matched for HR change during SCE test. Additional characteristics: height, weight, BMI, VO^2^ peak	HGS test study: 8 controls without DS (2♂) Age: 28 ± 3 years SCE test: 9 controls without DS (3♂) Age: 27 ± 3	Software: CM5 lead ECG (BIOPAC, Santa Barbara, CA) Sampling: 1000 Hz Duration: HGS test: 2 min periods: rest–HGS test (30% MVC)–recoverySCE test: two 6 min stages	Ambient temperature (22–24 °C)	Participants in the postprandial state for 4 h and refrained from caffeine and exercise for 24 h before data collection.	Participants encouraged to breath spontaneously without performing the Valsalva during HGS test.	HGS test—seated position; SCE test
Cunha et al., 2018 [63]	36 male DS subjects (3 groups):15 sedentary subjects Age: 26 ± 7 years 9 subjects with low intensity levels of PA Age: 26 ± 1 years 12 subjects with vigorous intensity levels of PA Age: 24 ± 2 years Additional characteristics: height, weight, BMI, lean and fat mass, IPAQ, MET	13 individuals without DS Age: 29 ± 4 years	Software: ECG (Wincardio Micromed 600 Hz, Brasilia, DF, Brazil) Sampling: 600 Hz Duration: 10 min	NR	NR	NR	Supine position with head elevation of 30°

DS—Down syndrome; NR—not reported; BMI—body mass index; ECG—electrocardiography; RER—respiratory exchange ratio; VE—minute ventilation; HRM—heart rate monitor; MVC—maximum voluntary contraction; VO^2^VT—oxygen uptake at ventilatory threshold; HGS—hand grip strength; PA—physical activity; IPAQ—International Physical Activity Questionnaire; MET—metabolic equivalent.

**Table 2:** Heart rate variability (HRV) measurement.

First Author and Year of Publication	Software	Artifact Correction	Time Series Length (Time/Beats)	Information about Data Normality	Time Domain Parameters (Units)	Frequency Domain Parameters and Bands (Units)	Frequency Analysis Method with Details	Nonlinear Parameters
Ferri et al., 1998 [49]	NR	10 min epoch ECG signals analyzed for automatic detection of R waves with a self-made program utilizing a simple threshold plus first derivative algorithm. Careful visual inspection for possible errors performed on all epochs.	10 min period within the first or second sleep cycle: W + S1 (sleep Stage 1, including wake around sleep), S2 (sleep Stage 2), SWS (sleep Stages 3 and/or 4) and REM sleep. The first 512 RR intervals from each epoch utilized for subsequent analysis steps.	NR	mRR (s), SDNN (NR), RMSSD (NR), pNN50 (%)	VLF <0.04 (s^2^/beat, cycles/beat), LF 0.04–0.15 Hz (s^2^/beat, cycles/beat), HF 0.15–0.4 Hz (s^2^/beat, cycles/beat), TP (s^2^/beat, cycles/beat), LF% (nu), HF% (nu), LF/HF	Parabolic interpolation used. FFT	Did not perform nonlinear analysis
Baynard et al., 2004 [50]	Heart Signal Co, Oulu, Finland	RR intervals visually inspected and filtered to eliminate undesirable noise or premature beats. Any RR interval that deviated > than 30% from the previous one was considered premature. Only recordings in which fewer than 2% of beats were filtered were included in HRV analysis.	Final 2 min of each submaximal stage, and the first 2 submaximal stages used for HRV analysis.	NR	SDNN (ms), RMSSD (ms^2^), pNN50 (%)	LF 0.04–0.15 Hz (ms^2^, nu), HF 0.15–0.40 Hz (ms^2^, nu), LF/HF Values in nu—graphical presentation	AR	Did not perform nonlinear analysis
Figuero et al., 2005 [51]	Heart Signal software (Oulu, Finland)	ECG data visually analyzed and edited for arrhythmias and artifacts.	HRV analyses performed for a 2 min period at rest, handgrip and recovery. Components detected from segments of 500 beats.	NR Parameters transformed to their natural logarithm for statistical analysis because of their skewed distribution.	Did not perform time domain analysis	LF 0.04–0.15 Hz (ms^2^) HF 0.15–0.40 Hz (ms^2^) LF/HF	AR (model order 10)	Did not perform nonlinear analysis
Iellamo et al., 2005 [52]	NR	NR	NR	Kolmogorov–Smirnov test	Did not perform time domain analysis	LF 0.03–0.15 Hz (ms^2^, nu) HF 0.15–0.40 Hz (ms^2^, nu)	The harmonic components of RR interval variability evaluated by the AR method (model order 8–12).	Did not perform nonlinear analysis
Goulopoulou et al., 2006 [53]	HEARTSTM, Finland	Visual and automatic editing to eliminate noise or premature beats. The filtering and analysis procedure: any time between heart beat interval that deviated > than 30% from the previous interval was considered premature. Recordings in which more than 2% of beats were filtered were repeated.	NR	Data not normally distributed. Logarithmic transformation was performed.	SDNN (ms) RMSSD (ms)	LF 0.04–0.14 Hz (ms^2^) HF 0.15–0.40 Hz (ms^2^) LF/HF TP	AR model (order 10)	Did not perform nonlinear analysis
Agiovlasitis et al., 2010 [54]	WinCPRS software (Absolute Aliens, Turku, Finland)	Ectopic beats and artifacts confirmed by visual inspection.	NR	NR	Did not perform time domain analysis	LF: 0.04–0.15 Hz (nu)HF: 0.15–0.40 Hz (nu) TP (ms^2^) LF/HF	From the blood pressure waves, the software generated the time series of the RR intervals analyzed to obtain the spectral components of HRV (AR).	Did not perform nonlinear analysis
Giagkoudaki et al., 2010 [55]	WinTer Holter Analyzer software	Ectopic beats and artifacts automatically and manually discarded.	NR	NR	SDNN (ms) SDANN (ms) SDNN index (ms) rMSSD (ms) pNN50 (ms)	LF: 0.04–0.15 Hz (ms^2^, nu) HF: 0.15–0.40 Hz (ms^2^, nu)	FFT The data were neither resampled nor interpolated.	Did not perform nonlinear analysis.
Agiovlasitis et al., 2011 [56]	WinCPRS software (Absolute Aliens, Turku, Finland)	RR intervals were steady-state and free of artifact and ectopy	550 continuous RR intervals	NR	mRR (ms)	Did not perform frequency domain analysis.	RR intervals detrended and resampled at 5 Hz.	ApEn (embedding dimension = 2, filter parameter = 20%), correlation dimension, StatAv - stationarity of the HR signal
Mendonca et al., 2011 [57]	(1) Polar Precision Performance Software (2) Kubios HRV Analysis Software 2.0	Visually inspected for undesirable premature beats and noise. RR interval interpreted as premature if it deviated from the previous interval by >30%.	256 consecutive RR intervals	Data tested for normality and homoscedasticity with the Kolmogorov–Smirnov and Levene’s tests	Did not perform time domain analysis.	LF: 0.04–0.15 Hz (ms^2^) HF: 0.15–1.00 Hz [ms^2^]	Time series detrended and resampled at 4 Hz. AR (model order 16).	Did not perform nonlinear analysis.
Mendonca et al., 2011 [58]	(1) Polar Precision Performance Software (2) Kubios HRV Analysis Software 2.0	Visually inspected for undesirable premature beats and noise.	256 consecutive RR intervals	Data tested for normality and homoscedasticity with the Kolmogorov–Smirnov and Levene’s tests	Did not perform time domain analysis.	Did not perform frequency domain analysis.	NR	DFA—short-term (4–16 beats) scaling exponent
Mendonca et al., 2013 [59]	(1) Polar Precision Performance Software (2) Kubios HRV Analysis Software 2.0	Analyses performed from the RR interval epochs free from ectopic beats and technical artifacts.	10 min	Data tested for normality and homoscedasticity with the Kolmogorov–Smirnov and Levene’s tests	Did not perform time domain analysis.	LF: 0.04–0.15 Hz (ms^2^, nu) HF: 0.15–0.40 Hz (ms^2^, nu) TP: 0.04–0.4 Hz	RR interval series resampled at 4 Hz (linear interpolation). A polynomial filter used to remove low frequency trends. AR (model order 16).	Did not perform nonlinear analysis
Bunsawat et al., 2015 [60]	HRV analyzed offline using Heart Signal software (Oulu, Finland)	ECG automatically and visually analyzed, edited for arrhythmias and artifacts.	RR interval variability evaluated from segments of 500 beats.	Shapiro–Wilk tests	RMSSD (ms)	LF: 0.04–0.15 Hz (ms^2^) HF: 0.15–0.40 Hz (ms^2^)	AR method (model order 10)	Did not perform nonlinear analysis
de Carvalho et al., 2015 [61]	(1) Polar Precision Performance SW software (2) Kubios	Manually complemented, visual inspection of the time series showed absence of artifacts.	1000 consecutive RR intervals	Shapiro–Wilks test	mRR (ms) SDNN (ms) RMSSD (ms) NN50 (ms) pNN50 (ms)	LF: 0.04–0.15 Hz (ms^2^, nu) HF: 0.15–0.40 Hz (ms^2^, nu) LF/HF	FFT	Did not perform nonlinear analysis.
Bunsawat et al., 2016 [62]	Heart Signal software (Oulu, Finland)	ECG automatically, visually analyzed, edited for arrhythmias and artifacts.	NR	Shapiro–Wilk tests	RMSSD (ms)	LF: 0.04–0.15 Hz (ms^2^) HF: 0.15–0.40 Hz (ms^2^)	AR method (model order 10)	Did not perform nonlinear analysis
Cunha et al., 2018 [63]	Kubios HRV 2.0 (Biosignal Analysis and Medical Imaging Group, Kuopio, Finland)	NR	5 min	Shapiro–Wilk tests	SDNN and RMSSD were chosen, in tables results for RR (ms) and total variability (ms^2^) provided	LF: 0.04–0.15 Hz (ms^2^, nu) HF: 0.15–0.40 Hz (ms^2^, nu) LF/HF	FFT Interpolation of 4 Hz, overlapped by 50%	Symbolic analysis: 0V, 1V, 2LV, 2UV

NR—not reported; ECG—electrocardiography; REM—rapid eye movement; mRR—mean RR interval; NN—intervals between normal R-peaks; SDNN—standard deviation of NN intervals; SDANN—standard deviation of the 5 min average NN intervals; RMSSD—root mean square successive difference; pNN50—percentage of adjacent NN intervals that differ from each other by more than 50 ms; HRV—heart rate variability; VLF—very low frequency; LF—low frequency; HF—high frequency; TP—total power; nu—normalized units; FFT—fast Fourier transform; AR—autoregressive model; ApEn—approximate entropy; DFA—detrended fluctuation analysis.

## Data Availability

Not applicable.

## Supplementary Materials

The following supporting information can be downloaded at: https://www.mdpi.com/article/10.3390/ijerph20020941/s1, Table S1: Results for heart rate (HR) and time-domain HRV parameters. Table S2: Results for frequency-domain and nonlinear HRV parameters.
