# Supplementary material for: Heart Rate Variability in Individuals with Down Syndrome: A Scoping Review with Methodological Considerations
Source: Int J Environ Res Public Health. 2023 Jan 4;20(2):941. doi: 10.3390/ijerph20020941 (PMC9859252; doi:10.3390/ijerph20020941)
Supplement: Supplementary file 1 [file ijerph-20-00941-s001.zip › Supplementary - Table S1.docx]

Table S1. Results for heart rate (HR) and time domain HRV parameters.

| **First author and year of publication** | **HR** | **Mean RR interval** | **SDNN** | **SDANN** | **RMSSD** | **pNN50** |
| --- | --- | --- | --- | --- | --- | --- |
| Ferri et al., 1998 [49] | NDR | stage W+S1 without sleep apnea 🡪 slow wave sleep (stages 3 and 4) without sleep apnea 🡪 (only for DS) stage 2 without apnea episodes 🡪 stage 2 with apnea episodes  Values in [s]  DS: 0.882 ± 0.200 🡪 0.916 ± 0.173 🡪 0.913 ± 0.178 🡪 0.904 ± 0.157  CG: 0.836 ± 0.236 🡪 0.934 ± 0.259 | stage W+S1 without sleep apnea 🡪 slow wave sleep (stages 3 and 4) without sleep apnea 🡪 (only for DS) stage 2 without apnea episodes 🡪 stage 2 with apnea episodes  No units provided  DS: 0.111 ± 0.053 🡪 0.086 ± 0.041 🡪 0.106 ± 0.056 🡪 0.151 ± 0.057  CG: 0.079 ± 0.046 🡪 0.074 ± 0.068 | NDR | stage W+S1 without sleep apnea 🡪 slow wave sleep (stages 3 and 4) without sleep apnea 🡪 (only for DS) stage 2 without apnea episodes 🡪 stage 2 with apnea episodes  No units provided  DS: 0.094 ± 0.069 🡪 0.087 ± 0.059 🡪 0.099 ± 0.081 🡪 0.099 ± 0.071  CG: 0.079 ± 0.058 🡪 0.097 ± 0.099 | stage W+S1 without sleep apnea 🡪 slow wave sleep (stages 3 and 4) without sleep apnea 🡪 (only for DS) stage 2 without apnea episodes 🡪 stage 2 with apnea episodes  Values in [%]  DS: 39.92 ± 21.77 🡪 45.04 ± 27.09 🡪 42.38 ± 26.31 🡪 37.02 ± 18.37  CG: 30.69 ± 22.32 🡪 42.01 ± 28.17 |
| Baynard et al., 2004 [50] | rest 🡪 peak  Values in [bpm]  DS: 71 ± 3 🡪 161 ± 5  MR: 78 ± 3 🡪 179 ± 5 | NDR | rest 🡪 submax 1 🡪 submax 2  Values in [ms]  DS: 66.1 ± 5.3 🡪 23.1 ± 3.2 🡪 21.5 ± 3.4  MR: 48.5 ± 5.5 🡪 31.1 ± 3.3 🡪 19.4 ± 3.5 | NDR | rest 🡪 submax 1 🡪 submax 2  Values in [ms^2^]  DS: 4422 ± 833 🡪 381 ± 207 🡪 396 ± 173  MR: 1499 ± 862 🡪 883 ± 214 🡪 344 ± 179 | rest 🡪 submax 1 🡪 submax 2  Values in [%]  DS: 32.8 ± 5.8 🡪 2.7 ± 1.3 🡪 1.4 ± 0.6  MR: 14.9 ± 6.0 🡪 2.0 ± 1.3 🡪 0.79 ± 0.6 |
| Figuero et al., 2005 [51] | rest 🡪 HGS test 🡪 recovery  Values in [bpm]  DS: 76 ± 3 🡪 80 ± 3 🡪 78 ± 3  CG: 78 ± 3 🡪 90 ± 3 🡪 77 ± 2 | NDR | NDR | NDR | NDR | NDR |
| Iellamo et al., 2005 [52] | NDR | rest 🡪 stand  Values in [ms]  DS: 1063 ± 62 🡪 932 ± 68  CG: 925 ± 41 🡪 726 ± 25 | NDR | NDR | NDR | NDR |
| Goulopoulou et al., 2006 [53] | Values in [bpm]  DS: 70 ± 2  CG: 66 ± 2 | NDR | Values in [ln ms]  DS: 4.12 ± 0.50  CG: 4.37 ± 0.09 | NDR | Values in [ln ms]  DS: 7.65 ± 0.15  CG: 7.72 ± 0.22 | NDR |
| Agiovlasitis et al., 2010 [54] | Graphical presentation of the data | NDR | NDR | NDR | NDR | NDR |
| Giagkoudaki et al., 2010 [55] | baseline 🡪 6 months  Values in [bpm]  DS: 78 ± 8 🡪 2.2% decrease  Baseline  CG: 71 ± 7 | NDR | baseline 🡪 6 months  Values in [ms]  DS: 152.8 ± 33.8 🡪 152.6 ± 33.7  Baseline  CG: 182.1 ± 27.5 | baseline 🡪 6 months  Values in [ms]  DS: 134.6 ± 30.8 🡪 137.5 ± 29.9  Baseline  CG: 163.9 ± 27.8 | baseline 🡪 6 months  Values in [ms]  DS: 40.8 ± 12.0 🡪 44.7 ± 10.6  Baseline  CG: 55.6 ± 16.6 | baseline 🡪 6 months  Values in [ms]  DS: 17.0 ± 10.1 🡪 24.0 ± 9.0  Baseline  CG: 28.5 ± 12.2 |
| Agiovlasitis et al., 2011 [56] | NDR | Graphical presentation of the data | NDR | NDR | NDR | NDR |
| Mendonca et al., 2011 [57] | Graphical presentation of the data | NDR | NDR | NDR | NDR | NDR |
| Mendonca et al., 2011 [58] | rest 🡪 treadmill exercise 🡪 recovery  Values in [bpm]  DS: 72 ± 3 🡪 increased 33 ± 2 🡪 decreased 28 ± 2  CG: 68 ± 3 🡪 increased 52 ± 4 🡪 decreased 46 ± 3 | NDR | NDR | NDR | NDR | NDR |
| Mendonca et al., 2013 [59] | pre-training 🡪 post-training  Values in [bpm]  DS: 63 ± 3 🡪 63 ± 2  NON-DS: 63 ± 3 🡪 62 ± 2 | NDR | NDR | NDR | NDR | NDR |
| Bunsawat et al.,  2015 [60] | Values in [bpm]  DS-not matched: 70 ± 3  DS-matched: 68 ± 2  CG: 62 ± 3 | NDR | NDR | NDR | Graphical presentation of the data | NDR |
| Dias de Carvalho et al., 2015 [61] | NDR | Values in [ms]  DS: 645.3, 610.8 – 679.7 (± 83.5)  CG: 661.4, 631.5-691.3 (± 72.5) | Values in [ms]  DS: 57.8, 50.8–64.9 (± 17.1)  CG: 48.7, 42.4–55.0 (± 15.3) | NDR | Values in [ms]  DS: 39.1, 33.5–44.6 (± 13.5)  CG: 33.9, 28.5–39.5 (± 13.3) | Values in [ms]  DS: 19.9, 14.7–25.1 (± 12.5)  CG: 13.8, 9.3–18.4 (± 10.9) |
| Bunsawat et al.,  2016 [62] | rest 🡪 HGS test  Values in [bpm]  DS: 68 ± 4 🡪 164 ± 5  CG: 80 ± 3 🡪 189 ± 4  rest 🡪 SCE test  Values in [bpm]  DS: 73 ± 3 🡪 167 ± 3  CG: 77 ± 3 🡪 189 ± 3 | NDR | NDR | NDR | rest 🡪 HGS test  Values in [ms]  DS: 53 ± 10 🡪 43 ± 7  CG: 36 ± 11 🡪 24 ± 8  rest 🡪 SCE test  Values in [ma]  DS: 31 ± 8 🡪 15 ± 3  CG: 41 ± 8 🡪 11 ± 3 | NDR |
| Cunha et al., 2018 [63] | NDR | Values in [ms]  SEDDS: 805 ± 43  LIDS: 952 ± 59  VIDS: 964 ± 59  Controls: 964 ± 182 | NDR | NDR | NDR | NDR |

HR–heart rate; mRR–mean RR interval; NN–intervals between normal R-peaks; SDNN—standard deviation of NN intervals; SDANN–standard deviation of the 5 minute average NN intervals; RMSSD—root mean square successive difference; pNN50—percentage of adjacent NN intervals that differ from each other by more than 50 ms; NDR–no data reported; DS–down syndrome; CG–control group; SCE–submaximal cycling exercise; SEDDS–sedentary subjects with DS; LIDS–low intensity levels of physical activity; VIDS–vigorous levels of physical activity
