# Supplementary material for: Heart Rate Variability in Individuals with Down Syndrome: A Scoping Review with Methodological Considerations
Source: Int J Environ Res Public Health. 2023 Jan 4;20(2):941. doi: 10.3390/ijerph20020941 (PMC9859252; doi:10.3390/ijerph20020941)
Supplement: Supplementary file 1 [file ijerph-20-00941-s001.zip › Supplementary - Table S2.docx]

Table S2. Results for frequency domain and nonlinear HRV parameters.

| **First author and year of publication** | **LF** | **HF** | **TP** | **LF/HF** | **Nonlinear parameters** |
| --- | --- | --- | --- | --- | --- |
| Ferri et al.1998 [49] | stage W+S1 without sleep apnea 🡪 slow wave sleep (stages 3 and 4) without sleep apnea 🡪 (only for DS) stage 2 without apnea episodes 🡪 stage 2 with apnea episodes  Values in [s^2^/beat]  DS: 3.09 ± 3.59 🡪 1.69 ± 🡪 1.92 🡪 3.01 ± 3.58 🡪 5.29 ± 4.87  CG: 0.99 ± 1.05 🡪 0.65 ± 0.91 | stage W+S1 without sleep apnea 🡪 slow wave sleep (stages 3 and 4) without sleep apnea 🡪 (only for DS) stage 2 without apnea episodes 🡪 stage 2 with apnea episodes  Values in [s^2^/beat]  DS: 2.79 ± 4.39 🡪 2.30 ± 2.49 🡪 3.60 ± 5. 47 🡪 3.15 ± 4.75  CG: 1.28 ± 1.33 🡪 4.64 ± 8.91 | stage W+S1 without sleep apnea 🡪 slow wave sleep (stages 3 and 4) without sleep apnea 🡪 (only for DS) stage 2 without apnea episodes 🡪 stage 2 with apnea episodes  Values in [s^2^/beat]  DS: 10.22 ± 11.78 🡪 5.14 ± 4.38 🡪 9.46 ± 8.67 🡪 17.10 ± 13.37  CG: 4.53 ± 4.80 🡪 5.54 ± 9.94 | stage W+S1 without sleep apnea 🡪 slow wave sleep (stages 3 and 4) without sleep apnea 🡪 (only for DS) stage 2 without apnea episodes 🡪 stage 2 with apnea episodes  DS: 1.73 ± 0.69 🡪 1.01 ± 0.72 🡪 1.11 ± 0.69 🡪 4.49 ± 4.78  CG: 0.91 ± 0.38 🡪 0.30 ± 0.22 | NDR |
| Baynard et al., 2004 [50] | rest 🡪 submax 1 🡪 submax 2  Values in [ms^2^]  DS: 1264 ± 226 🡪 204 ± 72 🡪 136 ± 49  MR: 885 ± 234 🡪 370 ± 75 🡪 141 ± 51 | rest 🡪 submax 1 🡪 submax 2  Values in [ms^2^]  DS: 1418 ± 269 🡪 115 ± 69 🡪 122 ± 58  MR: 580 ± 278 🡪 309 ± 71 🡪 103 ± 60 | NDR | rest 🡪 submax 1 🡪 submax 2  DS: 1.81 ± 0.42 🡪 2.88 ± 0.61 🡪 2.61 ± 0.95  MR: 2.28 ± 0.44 🡪 2.63 ± 0.63 🡪 3.09 ± 0.98 | NDR |
| Figuero et al.,  2005 [51] | rest 🡪 HGS test 🡪 recovery  Values in [ln ms^2^]  DS: 6.3 ± 0.2 🡪 6.2 ± 0.2 🡪 6.5 ± 0.2  CG: 7.2 ± 0.1 🡪 6.2 ± 0.2 🡪 7.1 ± 0.2 | rest 🡪 HGS test 🡪 recovery  Values in [ln ms^2^]  DS: 5.4 ± 0.2 🡪 5.1 ± 0.2 🡪 5.4 ± 0.3  CG: 5.6 ± 0.4 🡪 4.6 ± 0.4 🡪 6.3 ± 0.3 | NDR | rest 🡪 HGS test 🡪 recovery  DS: 1.18 ± 0.06 🡪 1.23 ± 0.06 🡪 1.24 ± 0.07  CG: 1.36 ± 0.08 🡪 1.44 ± 0.10 🡪 1.17 ± 0.05 | NDR |
| Iellamo et al., 2005 [52] | rest 🡪 stand  Values in [ms^2^]  DS: 1200 (650-2616) 🡪 498 (261-774)  CG: 853 (417-1263) 🡪 650 (252-1164)  Values in [nu]  DS: 51.3 (35.5-59.4) 🡪 64.3 (48.6-73.8)  CG: 53.6 (35.0-73.7) 🡪 82.7 (70.7-89.3) | rest 🡪 stand  Values in [ms^2^]  DS: 1102 (365-2216) 🡪 185 (131-237)  CG: 582 (323-1247) 🡪 121 (81-232)  Values in [nu]  DS: 34.6 (27.4-49.9) 🡪 18.7 (11.4-38.0)  CG: 38.1 (25.2-60.0) 🡪 14.9 (7.0-24.1) | NDR | NDR | NDR |
| Goulopoulou et al., 2006 [53] | Values in [ln ms^2^]  DS: 6.93 ± 0.12  CG: 7.26 ± 0.14 | Values in [ln ms^2^]  DS: 6.57 ± 0.16  CG: 6.65 ± 0.24 | Values in [ln ms^2^]  DS: 8.08 ± 0.11  CG: 8.44 ± 0.17 | DS: 0.35 ± 0.11  CG: 0.60 ± 0.17 | NDR |
| Agiovlasitis et al., 2010 [54] | Graphical presentation of the data | Graphical presentation of the data | Graphical presentation of the data | Graphical presentation of the data | NDR |
| Giagkoudaki et al., 2010 [55] | baseline 🡪 6 months  Values in [ms^2^]  DS: [24 h] 1127 ± 289 🡪 1165 ± 289  [day] 1124 ± 282 🡪 1156 ± 285  [night] 1142 ± 267 🡪 1114 ± 339  CG: [24 h] 1421 ± 276  [day] 1401 ± 327  [night] 1387 ± 231  Values in [nu]  DS: 66.3 🡪 67.8  CG: 68.9 | baseline 🡪 6 months  Values in [ms^2^]  DS: [24 h] 576 ± 322 🡪  686 ± 96  [day] 479 ± 271 🡪 590 ± 178  [night] 790 ± 6 🡪 1032 ± 120  CG: [24 h] 897 ± 279  [day] 716 ± 159  [night] 1343 ± 479  Values in [nu]  DS: 31.1 🡪 40.7  CG: 42.3 | NDR | Baseline 🡪 6 months  DS: 2.45 🡪 1.72  CG: 1.71 | NDR |
| Agiovlasitis et al., 2011 [56] | NDR | NDR | NDR | NDR | Graphical presentation of the data |
| Mendonca et al., 2011 [57] | Graphical presentation of the data | Graphical presentation of the data | NDR | Graphical presentation of the data | NDR |
| Mendonca et al., 2011 [58] | NDR | NDR | NDR | NDR | Graphical presentation of the data |
| Mendonca et al., 2013 [59] | pre-training 🡪 post-training  Values in [ln ms^2^]  DS: 6.6 ± 0.2 🡪 6.7 ± 0.2  NON-DS: 6.4 ± 0.3 🡪 6.3 ± 0.3 | pre-training 🡪 post-training  Values in [ln ms^2^]  DS: 6.1 ± 0.3 🡪 6.5 ± 0.3  NON-DS: 5.5 ± 0.3 🡪 5.7 ± 0.4 | pre-training 🡪 post-training  Values in [ln ms^2^]  DS: 7.3 ± 0.2 🡪 7.5 ± 0.2  NON-DS: 6.9 ± 0.3 🡪 6.9 ± 0.3 | pre-training 🡪 post-training  DS: 1.08 ± 0.03 🡪 1.04 ± 0.03  NON-DS: 1.17 ± 0.04 🡪 1.15 ± 0.08 | NDR |
| Bunsawat et al., 2015 [60] | rest 🡪 upright tilt  Values in [ms^2^]  DS-not matched: 1948 ± 755 🡪 1637 ± 934  DS-matched: 1304 ± 178 🡪 770 ± 154  CG: 1466 ± 289 🡪 1830 ± 377 | rest 🡪 upright tilt  Values in [ms^2^]  DS-not matched: 1155 ± 419 🡪 890 ± 364  DS-matched: 1308 ± 359 🡪 272 ± 312  CG: 1555 ± 359 🡪 825 ± 312 | rest 🡪 upright tilt  Values in [ms^2^]  DS-not matched: 6435 ± 1789 🡪 6188 ± 2243  DS-matched: 3995 ± 1646 🡪 2321 ± 2063  CG: 4461 ± 1532 🡪 4921 ± 1921 | rest 🡪 upright tilt  DS-not matched: 1.76 ± 0.49 🡪 4.10 ± 2.13  DS-matched: 2.27 ± 0.45 🡪 5.87 ± 1.96  CG: 1.69 ± 0.42 🡪 10.41 ± 1.82 | NDR |
| Dias de Carvalho et al., 2015 [61] | Values in [ms^2^]  DS: 1242, 917-1568 (± 788)  CG: 786, 588-985 (± 482)  Values in [nu]  DS: 69.1, 64.5-73.8 (± 11.3)  CG: 57.4, 52.5-62.2 (± 11.7) | Values in [ms^2^]  DS: 553, 410-696 (± 347)  CG: 589, 415-764 (± 423)  Values in [nu]  DS: 30.9, 26.3-35.5 (± 11.3)  CG: 42.5, 37.7-47.3 (± 11.6) | NDR | DS: 2.59, 2.14-3.05 (± 1.10)  CG: 1.58, 1.17-1.99 (± 0.99) | NDR |
| Bunsawat et al.,  2016 [62] | rest 🡪 HGS test  Values in [ms^2^]  DS: 1985 ± 568 🡪 1214 ± 327  CG: 1378 ± 635 🡪 693 ± 366  Values in [ln ms^2^]  DS: 6.99 ± 0.31 🡪 6.69 ± 0.30  CG: 7.11 ± 0.35 🡪 6.22 ± 0.33  rest 🡪 SCE test  Values in [ms^2^]  DS: 972 ± 284 🡪 127 ± 46  CG: 1679 ± 284 🡪 163 ± 46  Values in [ln ms^2^]  DS: 6.57 ± 0.22 🡪 4.32 ± 0.38  CG: 7.32 ± 0.22 🡪 4.72 ± 0.38 | rest 🡪 HGS test  Values in [ms^2^]  DS: 1461 ± 467 🡪 728 ± 185  CG: 604 ± 522 🡪 282 ± 207  Values in [ln ms^2^]  DS: 6.56 ± 0.46 🡪 5.97 ± 0.43  CG: 5.30 ± 0.52 🡪 4.76 ± 0.48  rest 🡪 SCE test  Values in [ms^2^]  DS: 359 ± 124 🡪 93 ± 29  CG: 343 ± 131 🡪 49 ± 31  Values in [ln ms^2^]  DS: 5.47 ± 0.33 🡪 3.67 ± 0.52  CG: 5.39 ± 0.35 🡪 3.13 ± 0.55 | rest 🡪 HGS test  Values in [ms^2^]  DS: 4432 ± 1212 🡪 3739 ± 705  CG: 3001 ± 1355 🡪 1964 ± 788  Values in [ln ms^2^]  DS: 7.96 ± 0.26 🡪 7.89 ± 0.28  CG: 7.86 ± 0.29 🡪 7.37 ± 0.31  rest 🡪 SCE test  Values in [ms^2^]  DS: 2175 ± 496 🡪 774 ± 146  CG: 3233 ± 526 🡪 556 ± 155  Values in [ln ms^2^]  DS: 7.50 ± 0.19 🡪 6.44 ± 0.24  CG: 7.98 ± 0.20 🡪 6.11 ± 0.26 | rest 🡪 HGS test  DS: 2.65 ± 1.78 🡪 2.98 ± 1.62  CG: 9.66 ± 1.99 🡪 6.55 ± 1.81  Values in [ln ratio]  DS: 1.08 ± 0.08 🡪 1.15 ± 0.08  CG: 1.42 ± 0.09 🡪 1.38 ± 0.09  rest 🡪 SCE test  DS: 4.76 ± 1.58 🡪 2.31 ± 5.27  CG: 8.60 ± 1.68 🡪 12.39 ± 5.59  Values in [ln ratio]  DS: 1.23 ± 0.07 🡪 1.26 ± 0.48  CG: 1.39 ± 0.08 🡪 2.22 ± 0.51 |  |
| Cunha et al., 2018 [63] | Values in [ms^2^]  DS:  SEDDS: 906 ± 258  LIDS: 469 ± 148  VIDS: 521 ± 135  CG: 964 ± 182  Values in [nu]  DS:  SEDDS: 70 ± 3  LIDS: 49 ± 4  VIDS: 45 ± 2  CG: 50 ± 4 | Values in [ms^2^]  DS:  SEDDS: 489 ± 332  LIDS: 482 ± 344  VIDS: 644 ± 233  CG: 831 ± 353  Values in [nu]  DS:  SEDDS: 30 ± 3  LIDS: 51 ± 4  VIDS: 55 ± 2  CG: 50 ± 4 | NR | DS:  SED DS: 2.87 ± 0.40  LIDS: 1.12 ± 0.21  VIDS: 0.88 ± 0.05  CG: 1.2 ± 0.20 | Values in [%]  DS:  SEDDS:  0V: 32 ± 4  1V: 43 ± 1  2LV: 8 ± 3  2UV: 16 ± 2  LIDS:  0V: 20 ± 5  1V: 45 ± 1  2LV: 9 ± 2  2UV: 23 ± 3  VIDS:  0V: 18 ± 3  1V: 42 ± 2  2LV: 9 ± 1  2UV: 28 ± 3  CG:  0V: 19 ± 3  1V: 46 ± 4  2LV: 15 ± 2  2UV: 19 ± 3 |

LF—low frequency; HF—high frequency; TP—total power; nu—normalized units; DS–down syndrome; CG–control group; MR–mental retardation; HGS–hand grip strength; SCE–submaximal cycling exercise; SEDDS–sedentary subjects with DS; LIDS–low intensity levels of physical activity; VIDS–vigorous levels of physical activity
